# Peer-led intervention to prevent and reduce STI transmission and improve sexual health in secondary schools (STASH): protocol for a feasibility study

**DOI:** 10.1186/s40814-018-0354-9

**Published:** 2018-11-29

**Authors:** Ross Forsyth, Carrie Purcell, Sarah Barry, Sharon Simpson, Rachael Hunter, Lisa McDaid, Lawrie Elliot, Julia Bailey, Kirsty Wetherall, Mark McCann, Chiara Broccatelli, Laurence Moore, Kirstin Mitchell

**Affiliations:** 1 MRC/CSO Social and Public Health Sciences Unit, University of Glasgow, Top Floor, 200 Renfield St, Glasgow, G2 3AX UK; 2 Research Department of Primary Care and Population Health, University College London, London, UK; 3 Department of Mathematics and Statistics, Strathclyde University, Glasgow, UK; 4 Department of Nursing and Community Health, School of Health and Life Sciences, Glasgow Caledonian University, Glasgow, UK; 5 Robertson Centre for Biostatistics, Institute of Health and Wellbeing, University of Glasgow, Glasgow, UK

**Keywords:** Sexually transmitted infections, Sexual health, Young people, Social media, Peer supporters, Social networks, Feasibility outcomes, Intervention development, Process evaluation

## Abstract

**Background:**

Young people in the UK are at highest risk of sexually transmitted infections and report higher levels of unsafe sex than any other age group. Involving peer supporters in intervention delivery is acceptable to students and effective in reducing risk behaviours via ‘diffusion of innovation’, particularly where peer supporters are influential in their networks. Informal peer-led interventions offer a useful alternative to peer-led didactic teaching, which has shown limited effects. Building on the successful ASSIST anti-smoking intervention, the ‘STis And Sexual Health’ (STASH) intervention involves identification and recruitment of the most influential students as peer supporters, training and support to these students by specialist trainers, positive sex and relationships messages, spread by peer supporters to their friendship groups in person and via social media.

**Methods/design:**

This protocol describes a feasibility trial of the STASH intervention in six schools. It builds on an earlier study phase of intervention co-development using patient and public involvement (PPI) activities, followed by a pilot of intervention components and evaluation tools in one school. Participants are fourth year (S4) students (aged 14–16) in state-funded Scottish secondary schools who have received some level of teacher-led sex education. The previous cohort of S4 students (those completing S4 in the year prior to the intervention) will serve as controls. Data will be collected from controls (month 16), baseline (month 20–21) and follow-up (month 27–30) via a web-based questionnaire, which will measure (and test the reliability of) primary outcome measures for a phase III trial (delayed initiation of/abstinence from sex and consistent condom use), secondary outcomes and mediators of sexual behaviour (including school climate and social networks). The main feasibility outcome is whether the study meets pre-set progression criteria regarding feasibility and acceptability, measured largely via a process evaluation (basic measures in all 6 schools and in-depth in 2-4 schools). An economic evaluation reporting costs alongside consequences will be conducted.

**Discussion:**

This study will inform decisions on the feasibility, design and sample size for a phase III effectiveness trial to assess whether the STASH intervention is effective in reducing the risk of sexually transmitted infections in young people.

**Trial registration:**

ISRCTN97369178

## Background

Young people (aged 16–24) in the UK report higher levels of unprotected sex than any other age group and are also at highest risk of sexually transmitted infections (STIs) [1]. STIs are associated with socio-economic inequality [1] and early intervention is required to prevent disadvantage converting to poor lifetime sexual health. Young people’s elevated risk has been linked to lack of awareness, insufficient knowledge of how to protect themselves, and social norms that denigrate safer sexual behaviour and undermine the quality of intimate relationships [2].

Students in school represent a ‘captive audience’ and schools are particularly well placed to reach disadvantaged young people at higher risk of adverse sexual health outcomes, and before sexual attitudes and behaviours become entrenched [3]. Young people who cite school as their main source of information about sex are less likely to report unsafe sex and previous STI diagnosis [4]. The proportion citing school as their main source is increasing [5], but the content and quality of provision of sex education in UK schools is variable [6]. Over two thirds of young people report inadequate knowledge at the time they first felt ready to have some sexual experience [5], suggesting significant room for improved delivery of school-based sex education.

Despite a significant literature, the effectiveness of peer-led approaches to improving sexual health among young people is equivocal [7–11]. Recent systematic reviews [9, 11] suggest that some rigorously evaluated interventions have shown improvements in knowledge, attitudes and intentions, but almost none have had an impact on behaviour. Harden et al. [12] identified five studies comparing the effectiveness of peer leaders to teachers in delivering the same intervention, of which two found peer leaders to be more effective than teachers. Peer leaders are thought to be less effective than adults at imparting factual information and getting students involved in classroom activities but more effective at establishing conservative (non-risky) norms [7]. Most studies rely on self-selection or teacher-selection, but both these strategies may result in educators who are not particularly credible and who find it difficult to reach high-risk students [13, 14]. On the other hand, in the effective anti-smoking intervention ‘ASSIST’ (A Stop Smoking In School Trial), the nomination of influential peers by students resulted in a diverse and representative group of peer leaders, despite initial doubts of students and staff [13]. The Students Together Against Negative Decisions (STAND) youth-focused sexual health intervention study also used peer-nomination and diffusion of social norm change (though not using social media), and the approach was both acceptable and effective [15]. These studies suggest that involving peer supporters in intervention delivery is acceptable to students and effective in reducing risk behaviours via diffusion of norms and attitudes [16], particularly where peer supporters are influential role models, chosen by peers. Interventions where peer supporters work informally within their social networks offer a useful alternative to peer-led didactic teaching. To our knowledge, there have been no European school-based studies in which peer supporters utilise social media to share sexual health messages.

As a health promotion tool, social media has strong potential and its use is rapidly gaining currency [17]. Two recent systematic reviews of social media interventions in sexual health [18, 19] found a significant impact on condom use and STI testing but with wide variation in effectiveness across study design. The studies included in these reviews mostly used text messaging or web-based interventions; only two used social networking sites (Facebook) and only one recruited existing social networks [20], though not networks within the same social system (such as a school). The reviews highlighted the importance of interactive and visually appealing content, maximising engagement and minimising burden [18, 19].

Prior to the STASH study, we conducted a patient and public involvement exercise (PPI) with 16 young people aged 14–19, which found strong support for a combination of the ASSIST model augmented by a social media component. PPI participants said that social media was fundamental to their everyday interactions with friends. They perceived sex education at school to be largely inadequate, expressed concern about discussing sexual matters with teachers and were enthusiastic about an intervention in which they could discuss sexual health with trained peers.

The overall aim of the STASH study is to develop and test the feasibility and acceptability of a school-based intervention delivered by peer supporters to prevent and reduce transmission of sexually transmitted infections and improve the sexual health of secondary school students aged 14–16 in the UK.

The development phase (described briefly below) is already complete. This protocol describes the methods and procedures for the feasibility trial and reflects our learning from the development phase of the study.

## Development of the STASH intervention

**Fig. 1:**
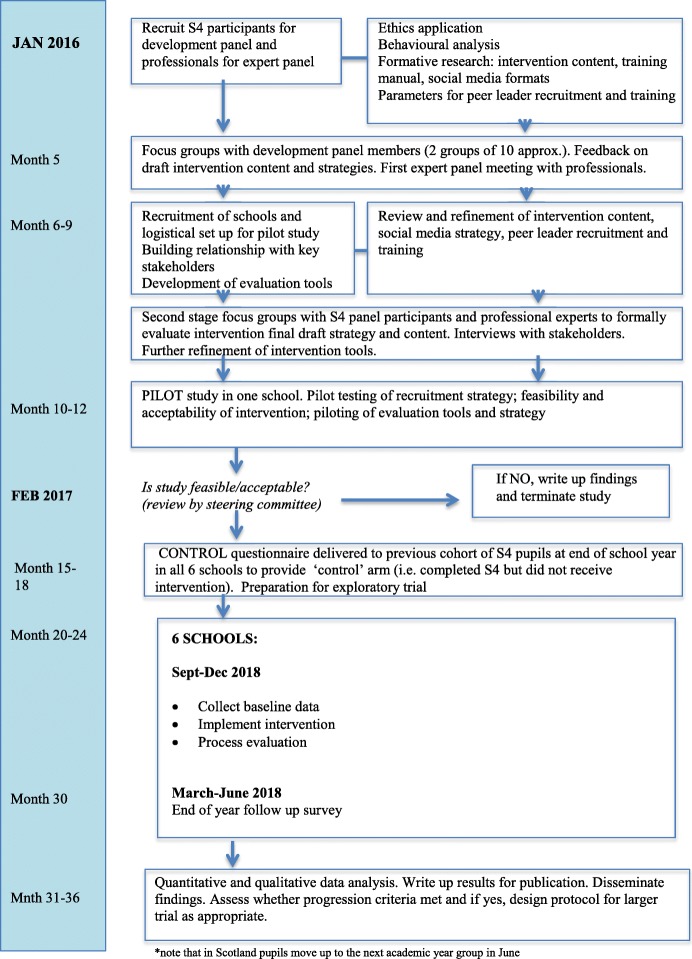
STASH study flow diagram

**Fig. 2:**
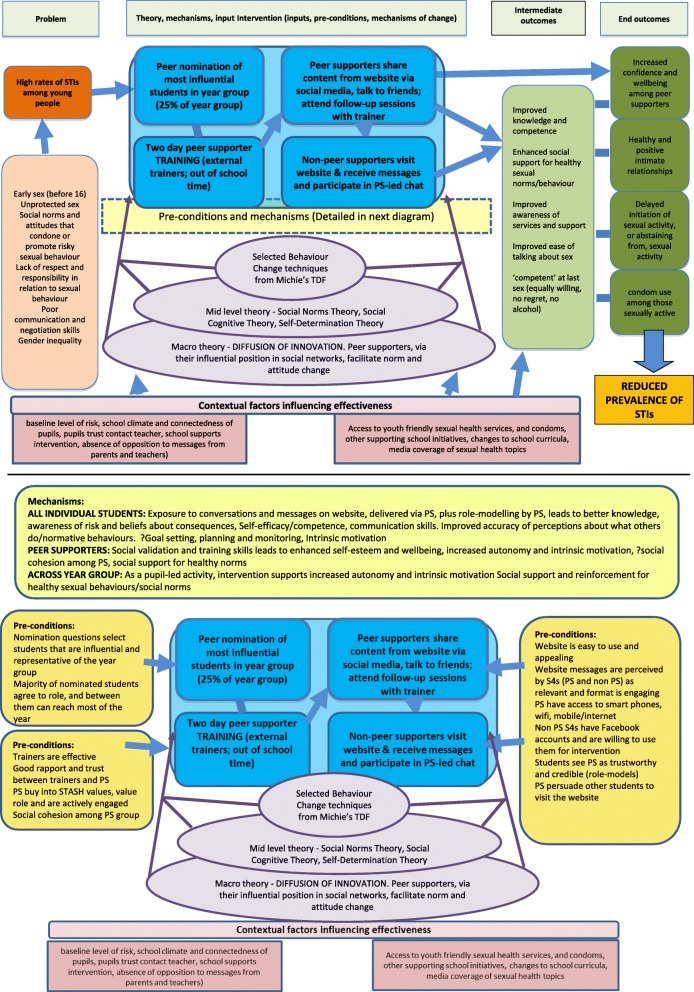
Study programme theory

Prior to this feasibility trial, we co-developed and formatively evaluated the intervention components and evaluation tools, piloted the intervention in one school and made refinements, following the UK Medical Research Council (MRC) guidance, [21] and the 6Squid approach [22]. Since key intervention components (peer nomination, two-day external training and follow-up sessions in school) were previously established by ASSIST [23], this stage focused on refinements to the intervention approach as required by the older age group, topic of sexual health, and use of social media as a means of communicating messages. The STASH study flow diagram (Fig. 1) illustrates the timeline of the development of the STASH intervention and the exploratory trial. In the development phase, we focused on understanding key sexual health issues and problem-drivers, identifying relevant behaviour change techniques, designing the website and training manual, identifying potential facilitators and barriers to intervention delivery, and testing and refining the programme theory (Fig. 2) and theoretical basis of the intervention.

Over a 15-month period, we undertook a scoping review of relevant peer-reviewed and grey literature; consultations with the target group (secondary students (aged 14 to 16 in non-study schools)); two development panel sessions with professionals in sexual health, education, youth work and social media; interviews with school teachers; a review of web-based resources both for young people and sexual health educators and a review of the functionality and appropriateness of candidate social media platforms. Both prior to and following the intervention pilot, we worked collaboratively with two youth training organisations (Fast Forward and West Lothian Drug and Alcohol Service) and a website developer (Antbits) to design training content and the STASH website.

We conducted a 1-month pilot in one school in months 10–12. This included a ‘dry run’ of intervention delivery, small-scale process evaluation, and piloting of the evaluation measures and tools. The results of the pilot were presented to an Independent Trial Steering Committee in month 15. They reviewed progress on intervention development, assessed the results of the pilot and, using clear progression criteria as a basis for the decision, recommended progression to stage two.

The STASH feasibility trial aims to establish the requirements to proceed to a full cluster randomised controlled trial of a peer-led intervention to prevent and reduce STI transmission and improve sexual health in secondary schools. The trial will be undertaken in six schools.

### Objectives for the feasibility trial

1. Assess the recruitment and retention of peer supporters, as well as feasibility, and acceptability of the intervention among peer supporters, participants and key stakeholders
2. Assess the fidelity and reach of intervention delivery by trainers and peer supporters, including barriers to, and facilitators of, successful implementation
3. Refine and test the programme theory (Fig. 2) and theoretical basis of the intervention
4. Enhance understanding of the potential of social media, when used by influential peers, to diffuse norm change and facilitate social support for healthy sexual behaviour
5. Determine key trial design parameters for a possible future large-scale trial, including recruitment and retention rates and strategies, outcome measures, intra-cluster correlation and sample size
6. Determine the key components of a future cost-effectiveness analysis and test data collection methods
7. Establish whether pre-set progression criteria are met and a larger scale trial is warranted

## Methods/design

We will conduct a feasibility trial in six schools, including process, outcome and health economic evaluation. The description of methods here conforms to the Standard Protocol Items: Recommendations for Interventional Trials (SPIRIT) guidelines for the reporting of study protocols.

### Setting and participants

Participants are fourth year (S4) students (aged 14–16). The intervention will be delivered in six state-funded secondary schools in Lothian region, Scotland. Faith schools will be invited to participate and included if they are willing to accept the intervention in full (including condom promotion as a strategy for STI prevention).

#### Inclusion criteria

All S4 students (aged 14–16) at state-funded schools who have received or are currently in receipt of teacher-led sex education, regardless of their sexual experience or individual level of risk are eligible for inclusion. 

#### Exclusion criteria

Private schools are excluded. 

### Sample size and selection

As a feasibility study, STASH is not designed to identify an estimate of effect and thus a standard power calculation is not appropriate. Allowing for non-response of 15% due to student absence, the sample size across all six schools is approximately 700 intervention participants and 700 controls. This should be sufficient to allow qualitative and quantitative progression criteria to be assessed and provide information on key parameters for the design of a future trial. It is premature to specify the required sample size for a future trial but is nevertheless useful to have a broad indication of the likely size of such a trial. It is anticipated that a full trial would be cluster randomised, with school as the unit of randomisation. Assuming a mean school year size of 150 students and an effect size of 0.1, then a trial of between 15 and 25 schools per arm would have 80% power across a range of scenarios in which (1) intra-cluster correlation is assumed to be either 0.01 or 0.03, (2) there is one or two primary outcomes, and (3) follow-up rates vary between 75% and 85%.

Schools will be recruited via local authority education services. STASH project staff will initially contact school management teams by email and meetings will be arranged with those expressing interest. Follow-up emails and calls will be made as necessary. Response rates, and any stated reasons for non-participation, will be recorded. Schools will be recruited on a first come, first served basis, while keeping mindful of the need to ensure variation in terms of school size, geographic location and area deprivation.

The feasibility of recruitment of schools to cluster randomised trials has been well established in many previous trials, so we have opted to implement the intervention in all six study schools, rather than recruit a larger number of schools and randomise half to the control. The prior cohort of S4 students (i.e. those completing S4 in the year prior to the intervention) in each school will complete the outcome questionnaire towards the end of their academic year (and prior to implementation of the intervention in the subsequent cohort of S4 students) and their data will be compared with the cohort of S4 students participating in the intervention. Therefore, the prior cohort of S4 students will serve as controls who received usual provision of sex and relationships education, which in most Lothian schools is a version of the Sexual Health And Relationships Education (SHARE) package [24].

Schools will be considered formally recruited to the study on the administration of the control questionnaire and signature of the research contract by a senior staff member. Any school opting to discontinue with the study after this point will be considered a withdrawal.

To maximise retention, schools will be given £500 to compensate for disruption to school life due to the questionnaire and process evaluation activities, plus £500 to compensate for staff time taken up by intervention activities (e.g. staff attendance at training).

### The intervention

The STASH intervention is a school-based and peer-led intervention in which influential peers disseminate positive sex and relationships messages via personal conversations and social media. It builds on an effective peer-led smoking prevention intervention (ASSIST) in secondary schools, which recruited and trained ‘influential’ students (aged 12/13) as peer supporters to spread and sustain non-smoking norms through informal interactions with peers [23]. Like ASSIST, the STASH intervention is underpinned by ‘Diffusion of Innovation’ Theory [16], which suggests that over time, novel ideas and behaviours spread through members of a social system via communication channels, with peer supporters serving as ‘early adopters’ or innovators’. The STASH intervention also draws on a range of behaviour change theories in the design of intervention content and messages (e.g. Social Norms Theory (SNT) [25], Social Cognitive Theory (SCT) [26], Self-Determination Theory (SDT) [27]). The STASH intervention differs from ASSIST in several key ways: a focus on sexual health, an older age group (14 to 16) and the use of social media in addition to face-to-face interactions.

The intervention will be delivered in five stages**:**

1. Nomination of peer supporters. All students in fourth year (Scottish S4; aged 14–16) will be asked to complete a peer nomination questionnaire. We will use the same three questions used in ASSIST (Who do you respect in S4 at your school? Who are good leaders in sports or other group activities in S4 at your school? Who do you look up to in S4 at your school?’), plus several new recruitment questions. The 25% of young people receiving the most nominations, stratified by gender, will be invited to a recruitment meeting
2. Recruitment of peer supporters. A meeting will be held with nominees in each school, in which the trainers explain the purpose of the intervention and the role of peer supporters and answer questions. The aim is to recruit between 15 and 20% of the entire year group to the role. If attendance at the recruitment meeting is poor, or if role uptake low or skewed significantly towards one gender, a second recruitment meeting may be held. Letters (information and consent forms) will be sent to parents/carers of all nominated students expressing interest in participation. Peer supporters are then asked to provide their consent, and that of their parents, to their participation in training and the peer supporter role
3. Two-day peer supporter training in school time and at a venue outside of school. The training will be facilitated by Fast Forward and West Lothian Drug and Alcohol Service. It aims to equip peer supporters with knowledge, skills and confidence required for the role, build motivation and enthusiasm for the role, and generate trust and rapport within the peer supporter group and between peer supporters and trainers. Drawing on the STASH theory of change, the training activities will focus on building sexual health knowledge and skills, understanding consequences and risk, building self-esteem and self-efficacy, reinforcing social support for healthy sexual norms, (competence), boosting intrinsic motivation and autonomy. Peer supporters will also be trained in skills required for the role such as listening and influencing skills, identifying and responding to sensitive disclosures and signposting to sources of help. During the training, peer supporters will sign a code of conduct and will agree a plan to ‘announce’ the project to their year group (an assembly, video, bulletin are all options)
4. Peer support work. The period in which peer supporters are active in their role is between 5 and 10 weeks. Activities will include
  - Peer-delivered activities centre on education and persuasion to target determinants of behaviour identified in the behavioural analysis. Peer supporters will establish a ‘secret’ Facebook group (invite-only groups; highest privacy setting), comprising their friends and the STASH trainer. They will be encouraged to paste messages and links from the STASH website to this group and to initiate face-to-face conversations centred on STASH messages. They will be encouraged to alert their friends to online and local sources of support and help. To ensure maximum reach, peer supporters will use ‘STASH cards’ to advertise a non-sharing version of the STASH website to students beyond their immediate friendship group and/or who are not members of their secret Facebook group. Peer supporters are supported by a trainer, as well as an appointed contact teacher. As far as possible, peer supporters will be able to engage with intervention resources flexibly, for instance, they can choose which messages and links to share and have the option of editing messages
  - Trainer-led activities include moderation of threaded discussions and monitoring of content, communication with peer supporter and facilitating face-to-face follow-up meetings (weekly or fortnightly) with the peer supporters group
5. Acknowledgment. Peer supporters who complete the role will be provided with University of Glasgow certificates and, if they complete the online questionnaire, £10 gift vouchers. Schools may also support peer supporters towards the attainment of a credited Youth Achievement Award. Our PPI work suggested external recognition provides a strong incentive

Retention of peer supporters will be measured by role completion, defined as the proportion of peer supporters who attend both days of training, attend at least two follow-up meetings and send at least three messages. We will employ a range of strategies to maximise peer supporter retention including developing high-quality intervention materials, building collegiate and open relationships between project staff and school staff and building open and supportive relationships between trainers and peer supporters, using existing class sessions (rather than student personal time) for intervention activities.

### Outcome measures and data collection

The main feasibility study outcome is whether the study meets pre-set progression criteria regarding the feasibility and acceptability of the intervention and evaluation methods. Table 1 outlines the progression criteria.

**Table 1:** STASH study criteria for Progression to stage III Randomised Controlled Trial

	Criteria	INDICATOR and TARGET	Recommendation if GREEN, AMBER or RED target met	METHOD OF ASSESSMENT	RATIONALE
1	Was it feasible to implement STASH in 4 of 6 schools?	GREEN: In each of 4 schools, 60% of nominated students are recruited and complete the training	Very strong indication to proceed	Project monitoring data	Based on learning from ASSIST, 60% is estimated as the proportion required to ensure that peer supporters are representative and reach across the entire year group.
		AMBER: In each of 4 schools, 50% of nominated students are recruited and complete the training	Medium indication to proceed. Discuss with Trial Steering committee (TSC) and proceed with identified plan to improve performance on indicator in Phase III trial		
		RED: Amber target achieved in fewer than 4 schools	Indication of doubt as to whether to proceed. Discuss with TSC, and only proceed if other indicators are amber/green and there is a clear mitigating strategy		
2	Was STASH acceptable to peer supporters in 4 of 6 schools?	GREEN: In each of 4 schools 60% of peer supporters who complete the training, send three or more messages/have three or more conversations, and attend two or more follow-up meetings and 60% of peer supporters report that they ‘liked’ the role	Very strong indication to proceed	Facebook monitoring data Peer Supporter Questionnaire	We consider 60% a reasonable target given the sensitivity of the topic and challenge involved for peer supporters. 60% represents a majority while not providing an over-ambitious target, given that the intervention is new to schools and not institutionally embedded. We would expect role acceptability to increase with further iterations (e.g. in a full RCT) which lead to greater clarity and institutional support. We view 60% as a ‘starting point’ for this feasibility stage.
		AMBER: In each of 4 schools 50% who complete the training send three or more messages/have three or more conversations, and attend two or more follow-up meetings and 45% like role	Medium indication to proceed. Recommend as per amber target for Criteria 1		
		RED: Amber target achieved in fewer than 4 schools	Indication of doubt as to whether to proceed. Recommend as per red target for Criteria 1		
3	Was STASH acceptable to stakeholders and target group?	GREEN: In each of 4 schools, 60% of students who are exposed to STASH agree that the intervention was acceptable. No major acceptability issues raised by participating schools (identified via process evaluation or communication with school) Less than 15% of peer supporters report that parents were unhappy about them being a peer supporter	Very strong indication to proceed	Follow-up Questionnaire Process evaluation interviews Peer Supporter questionnaire	We consider 60% a reasonable target given the sensitivity of the topic. 60% represents a majority and is realistic in the context of a feasibility study. Acceptability to teachers and school leadership will be assessed qualitatively, hence a focus on identification of major issues rather than a quantitative target.
		AMBER: In each of 4 schools, 50% rate intervention as acceptable Less than 20% of peer supporters report that parents were unhappy about them being a peer supporter One or two major acceptability issues raised by participating schools but mitigating strategy identified	Medium indication to proceed. Recommendation as per amber target for Criteria 1		
		RED: Amber target achieved in fewer than 4 schools Major acceptability issues raised by schools with no possible mitigating strategy	Indication of doubt as to whether to proceed. Recommendation as per red target for Criteria 1		
4	Were the evaluation methods acceptable and feasible?	GREEN: In each of 4 schools, student response rates of >70% at baseline and follow up	Very strong indication to proceed	Baseline and Follow-up Questionnaires	We consider a response rate of 70% sufficient to undertake analysis, and feasible given that this cohort are undertaking public examinations at the end of the year. Response rates in the pilot were lower than expected for a school survey. Parental opt out has been very low and nearly all students in attendance complete the questionnaire, but due to the age group (and linked to area deprivation) there are students who are regularly absent (e.g. because they also attend other services).
		AMBER: In each of 4 schools, student response rates of >60%	Medium indication to proceed. Recommendation as per amber target for Criteria 1		
		RED: Amber target achieved in fewer than 4 schools	Indication of doubt as to whether to proceed. Recommendation as per red target for Criteria 1		

A key aim of the study is to identify suitable measures for primary and secondary outcomes and mediators to take forward in a phase III trial. The end outcomes are delayed initiation/abstinence from sexual activity and consistent condom use among those who are sexually active. We will consider a range of potential primary outcome indicators including condom use at last vaginal intercourse, condom/dental dam use at last oral sex, number of sexual partners in last 3 months, number of sexual partners in last 3 months with no condom use, frequency of condom use in the last 6 months, proportion of students who have not had sex in past 6 months or have not yet had sex at all.

We will assess the feasibility and acceptability of longer-term follow-up, including linkage to routine NHS data on STI diagnosis and use of sexual health services, subsequent to intervention exposure. We will explore this via discussion with colleagues with expertise in data linkage and via an item on acceptability to students in the questionnaire.

Informed by our theory of change, we will investigate a range of potential secondary outcomes. Measures for the survey include validated scales, items from validated scales and items from existing sexual health intervention evaluations (e.g. RIPPLE [28]; SHARE [24]) and surveys (e.g. Natsal [29]). Measures are as follows:

- STI prevention and sexual health-related knowledge (drawn from, and adaptations of, existing survey items)
- Ease of talking about sex with parents and friends (adapted Natsal measure)
- Confidence in STI prevention skills (adapted from range of existing survey items)
- ‘Competence’ at intercourse based on the four-item Natsal Sexual Competence measure which includes items on willingness, acceptability of timing, autonomy and use of contraception. The aggregate score represents the extent to which first intercourse was competent in a public health sense [30]
- Sexual attitudes and adherence to sexual health norms (12 new items, adapted from range of existing survey items)
- Perception of whether others are sexually active
- Self-reported quality of intimate relationships. Seven newly designed items
- Distress about sex life (Natsal survey item)
- Self-reported use of internet and social media for finding sexual health information, sexting and viewing sexual images online (six new items)
- Short Warwick-Edinburgh Mental Well-Being Scale (SWEMWBS). A seven-item scale measuring a broad concept of positive emotional well-being including psychological functioning, cognitive-evaluative dimensions and affective-emotional aspects [31]. Aggregate scores form a ‘well-being index’ with a higher score representing greater well-being
- Conversations about STASH-related topics (new items for STASH)
- Self-esteem (two items from RIPPLE questionnaire, plus single-item global measure [32]
- Self-reported sexual activity
- Knowledge of local sexual health services
- Self-reported health-related healthcare resource use (STI testing and treatment, contraception including out of pocket costs)
- Child Health Utility 9 dimensions (CHU9D) as a measure of the quality of life and associated algorithm for calculating quality-adjusted life years (QALYs) [33]

#### Effect modifiers

- Single-item measures of gender, socio-economic status (IMD, free school meal status), ethnicity, educational attainment (qualifications being studied and intention to leave school) and religiosity.
- Self-reported risk behaviours in peer group (four items from existing surveys)
- School climate and engagement (measured using selected items from the Beyond Blue ‘school climate’ scale, [34]),
- Parental monitoring. Three new items.
- Sexual attraction and identity (modified version of the Kinsey Scale; item from Natsal)
- Self-regulation. Three items drawn from the 36-item Adolescent Self-Regulatory Inventory [35]
- Importance of social media to social life (two newly designed items)
- Social network questions, asking about up to six friends (whether or not in the same year group, time spent together online and offline, comfort in sharing something private)
- Exposure to intervention activities and messages.

#### Outcome data collection

The intervention will be delivered to the S4 student cohort in all six schools between month 20 (August 2017) and month 30 (June 2018), as outlined in the STASH study flow diagram (Fig. 1). All measures will be collected in month 15–18 (March to June 2017) from the previous S4 cohort in each of the six schools who are unexposed to the intervention (since they will have progressed to S5 by the time the intervention is delivered). Collecting data from this previous cohort doubles the information we have to estimate student consent and response rates and evaluate outcome measures, questionnaire content and data collection procedures. It also provides repeated cross-sectional data to assist in the estimation of potential intervention effects. Since both control and intervention students will complete measures in March or June (final month before exams start or first month after exams end), though 1 year apart, their data can be considered comparable.

The baseline/follow-up questionnaire was cognitively tested at stage one via six interviews with students attending the development panels to test comprehension and acceptability of items. Data from the pilot was analysed to check for possible misunderstanding, spread of responses and item-total correlations. The baseline and follow-up questionnaires will be administered as a web-based survey by trained fieldworkers under ‘exam conditions’. ‘Mop up’ visits will be scheduled to collect data from absentees.

### Economic measures and data collection

The aim of the economic evaluation is to assess the feasibility and acceptability of collecting sexual health-related healthcare resource and quality of life (QoL) data to inform the design of a cost-effectiveness analysis to be undertaken alongside a full randomised control trial (RCT). Self-reported health-related healthcare resource use (STI testing and treatment, contraception including out of pocket costs) will be collected via the baseline and follow-up questionnaires and costed using published sources. The CHU9D [36] will be used to calculate quality-adjusted life years (QALYs). In addition, we will undertake a detailed cost analysis of the intervention. This will include time taken in the identification and training of peer supporters and any additional time spent by teachers on the STASH intervention.

### Process measures and data collection

The process evaluation will assess possible mechanisms of impact, feasibility and acceptability of the research design and conduct, implementation and contextual dependence [22]. This involves examination of acceptability of intervention form and content; reach; exposure; fidelity (the extent to which the intervention delivered as intended); contextual factors including barriers to, and facilitators of, implementation; factors affecting recruitment and retention; relevance to the target group; and perceived impact of the intervention. The process evaluation will contribute to developing the STASH programme theory by exploring whether and how norm change and social support for healthy sexual behaviour is spread through a relatively closed social system, identifying which components are potentially most important to the intervention’s success and providing possible explanations for components that did not work so well.

Feasibility study process evaluation work will involve basic evaluation activities in all six schools and in-depth evaluation activities in two to four ‘case study’ schools. Case study schools will be selected on the basis of school location/population served (urban/semi-urban), school size and proportion of free school meals.

Basic evaluation activities will comprise the following: (1) quantitative student evaluation of two-day peer supporter training; (2) interviews with all three trainers; (3) online questionnaire to be completed by all trained peer supporters focusing on reasons for engagement/non-engagement, preferred communication approaches, perceived challenges and factors facilitating role, perceived response of peers; (4) social network analysis (data from baseline and follow-up questionnaire and Facebook group membership); (5) project monitoring log capturing information including percentage and characteristics of those completing training/accepting role/formally withdrawing, field notes on relevant meetings and relevant contemporaneous events; (6) peer supporter activity log (on group membership, posting activity, face-to-face conversations); (7) analysis of relevant items from the follow-up questionnaire on exposure and effect modifiers; (8) fieldwork/trainer observation pro formas for individual intervention delivery and data collection sessions.

In-depth evaluation activities will include the above with the addition of (9) structured observation of peer supporter training to assess engagement by peer supporters and document reactions to the intervention, (10) friendship group interviews with trained peer supporters (one to two groups per school), (11) paired/group interviews with non-peer supporter S4 s (one to two groups per school) and (12) individual interviews with teachers (two per school).

### Data analysis

#### Evaluation feasibility and acceptability outcomes

The feasibility and acceptability outcomes that constitute the progression criteria (Table 1) will be summarised overall and (anonymously) by the school.

The data linkage acceptability question will be summarised overall and by key factors at baseline (such as socio-demographic, sexual experience).

#### Baseline characteristics

Baseline characteristics (including deprivation) will be summarised overall and by study group (intervention/control) and for those who were and were not followed up (or dropped out of intervention). Exploratory investigations of the associations between baseline characteristics and successful follow-up may be carried out using appropriate statistical tests and mixed effects logistic regression models (to reflect the clustered nature of the data by school), to identify potential sources of bias in future studies.

#### Outcome data

Outcome data will be summarised overall and by study group. Mixed-effects regression models, with a random effect for school, will be used to estimate the magnitude of intervention effects with 95% confidence intervals. The effects of individual-level factors (effect modifiers) potentially associated with outcomes will be explored by extending these regression models, to identify key trial design parameters in future studies. Interaction models may be considered to explore whether the intervention might be more or less effective (or inferior) for particular subgroups of students. The within-school intra-class correlation coefficients will be reported for each outcome and used to inform sample size calculations for the future RCT. The study is powered for exploratory analysis only and so analyses will not be testing hypotheses regarding intervention effectiveness. Missing data will not be imputed since this is a feasibility study.

#### Analysis for economic evaluation

We will conduct an initial cost-consequences analysis reporting descriptive statistics for the exposed and unexposed groups separately. Statistics will include data completeness and percentage that each resource contributes to total costs. This will inform areas where additional information may be needed in a full trial for more accurate costings. Costs will include the cost of peer supporter training and teacher time associated with STASH in the intervention group and sexual health resource use (STI testing, treatment and contraception) multiplied by published unit costs for both groups. QALYs will be calculated as the area under the curve using student responses to the CHU9D at the two time-points. The responsiveness of the CHU9D to the primary outcome and other hypothesised mechanisms of change will also be evaluated. Descriptive statistics will be reported for questionnaire completion alongside means, standard deviations and bootstrapped 95% confidence intervals by exposed/unexposed group for each variable**.** This will form the basis of a cost-consequences analysis, reporting costs alongside consequences such as QALYs and measures of behavioural change for intervention and control groups. As per the outcome analysis, missing data will not be imputed.

#### Qualitative analysis

The qualitative analysis will take a thematic analytic approach informed by the Framework method [37]. A focused coding framework will be developed and applied to interview transcripts and ethnographic observational notes. The framework will be strongly informed by the programme theory as well as the aims of the feasibility trial (fidelity, acceptability, exposure, reach, context, recruitment and retention), while also mindful of emerging issues not anticipated by the research team.

Following this categorising stage, the data will then be examined across sources (students, teachers, trainers) and cases (schools), to explore commonalities and differences in accounts of the trial, to develop potential explanations for these and to better understand the functioning of the intervention. A subsequent integrative analysis will be conducted to bring together key components of the qualitative and quantitative data. Integrative analysis involves placing all relevant data in one integrative matrix and assessing for synergy and will focus on assessing the overall feasibility of the intervention.

#### Social network analysis

Social networks exert important influences on health behaviour, including among adolescents [38] and we wish to contribute to the understanding of how these networks operate. These data will enable the identification of the extent of friendships outside the school, and therefore estimate potential contamination in a full-scale cluster randomised trial, and will assess the reach of the peer supporters across the S4 year group. Social network analysis will provide us with new ways of investigating how offline and online interactions are shaped and sustained by relational mechanisms. In particular, they will enable identification of clusters, of individuals considered particular trustworthy, of ‘information hubs’ (measures of centrality [39]), as well as isolated individuals (no nominations). The analysis will help to highlight whether perceived trustworthiness overlaps with the peer supporter role (are the most trusted individuals also the most active peer leaders?). We plan to investigate homophily, i.e. whether subgroups of the network are formed around gender (e.g. do girls only talk to girls?), sexual behaviour (do high-risk individuals mainly talk to other high-risk individuals?), norms (are those who voice specific opinions largely connected to others with similar opinions?) and other node characteristics.

#### Participant information and informed consent

A senior staff member from participating schools will be asked to sign a research contract outlining the responsibilities of the school and the researchers. Prior to the start of the intervention phase, S4 students and their parents will receive an information sheet telling them about the STASH study. Informed opt-in consent from the students will be required for all specific components of the research study, including questionnaires (opt-out for parents), interviews and focus groups and from the peer supporters and their parents for them to participate in the training and take on the peer supporter role. Parental opt-out consent for the questionnaire is important since research over many years shows that the lower participation with opt-in consent is strongly biased away from the most vulnerable young people [40].

At all study stages, participants will be informed that they can withdraw from any research component at any time without prejudicing their experience at school. Research participants will be reassured that their answers will be treated in confidence. Researchers will only break confidentiality if a disclosure in a face-to-face interview suggests that a young person might be at risk of serious harm or of harming others. The intervention website will include links to local and national referral services.

#### Procedures for reporting harms and safeguarding issues

The STASH research team will employ strategies to minimise the likelihood of untoward incidents with potential to cause harm. This includes behavior such as inappropriate/inaccurate posting, online bullying and breaches of private information by peers.

At the training, peer supporters will be required to sign up to a code of conduct (the STASH Charter). Social media use will be confined to private (‘secret’, ie. non-visible, invite-only) Facebook groups, of which a STASH trainer will be a member. Trainers will conduct monitoring ‘spot checks’, and peer supporters will also be encouraged to report any untoward incidents promptly to the trainer and/or STASH contact teacher. Students will also have the option to privately message the trainer as required. In line with STASH study procedure for reporting harms, the trainer should pass on any concerns to the contact teacher without delay. Procedures for dealing with disrespectful/aggressive online behaviour will follow the information and communication technologies (ICT) code of conduct and discipline code of participating schools.

Appropriate responses to sensitive disclosures and procedures for reporting potential child protection issues will be explained—and their importance emphasised—during the 2-day peer supporter training. In particular, peer supporters will be advised on how to respond to sensitive information shared by peers (including respecting privacy) and when to share these with the STASH contact teacher/Designated Member of Staff (DMS). Peer supporters will be advised that any disclosures about which they feel worried or uncomfortable should be reported without delay to the contact teacher/DMS. Schools have in place procedures for handling disclosures relating to child protection, which should then be followed as normal. Peer supporters are advised that they may also contact the STASH trainer via Facebook, if they wish (see below).

The STASH contact teacher/designated staff member and those delivering the intervention (STASH trainers and peer supporters) will be asked to notify the research team within five working days if any harm occurs to a member of staff or student, as a direct result of taking part in the STASH trial. Members of the research team will be required to document any harms reported to them during trial data collection. These will be discussed with the principal investigators, triaged according to severity and reported the Trial Steering Committee and funders as appropriate.

#### Data monitoring and quality assurance

The project is overseen by an Independent Trial Steering Committee (TSC), consisting of a Chair, and three other members. The co-principal investigators (LM, KM) are non-independent members. The TSC met at month 15 to approve progression from stage 1 to stage 2 from pilot to exploratory trial and will also meet to review the results of the feasibility stage of the trial. The TSC provides overall supervision for the study and advice through its independent chair. The project will use standardised research protocols and adherence will be monitored by the Project Executive Group, Trial Management Group and TSC. Since the study is low risk, non-randomised and does not include interim analysis, a Data Monitoring and Ethics Committee (DMEC) is not required. The TSC will be asked to cover the functions of the DMEC, in particular in relation to ethical issues, monitoring of any unintended outcomes and the continuation of the trial.

### Data management

The confidentiality of participants will be protected in accordance with the Data Protection Act 1998. A unique identifier will be given as soon as possible to data transcripts and questionnaires. Personal details will be removed and stored separately. A de-code key to the ID will be kept secure and separate from the electronic data. Digital recording of interviews will be stored on an encrypted and password protected computer (network drive), separately from identifying information. Transcripts printed for the purpose of analysis will be stored in a locked cabinet. At the end of each day, they will be returned to the locked cabinet.

Access to data will be restricted to the research team and a transcription service with whom the University of Glasgow has an ongoing contractual arrangement, confidentiality agreement and relationship of trust. Data sent off-site for transcription will be logged in and out and encrypted data will be sent via secure website transfer and with support from the MRC/CSO Social and Public Health Sciences Unit IT team.

All data will be kept for at least 10 years in line with University of Glasgow Research Governance Framework Regulations for clinical research. Data will be stored confidentially on password-protected servers maintained on the University of Glasgow network. Data integrity will be checked every 2/3 years. We will make anonymised annotated qualitative extracts plus raw quantitative data available to other researchers on request and will deposit the data in an appropriate database, such as the UK Data Archive. Further details on data management are described in a separate Data Management Plan.

In reporting the results of the interviews and focus groups, care will be taken to use quotations which do not reveal the identity of respondents or schools. All data collected as part of the project will be treated as confidential and will only be viewed by members of the research team; anonymised data will be used wherever possible. All procedures for data storage, processing and management will comply with the Data Protection Act 1998.

### Dissemination

We will aim to submit at least three academic papers on the study to relevant and highly regarded journals. We will present interim results at academic conferences such as the Sunbelt Social Networking conference and UK Society of Behavioural Medicine. We will hold a dissemination meeting for key policy leads and practitioners, including representatives from youth, educational and sexual health charities, as well as the Scottish Government. We will seek invitations to speak at relevant stakeholder meetings and conferences such as Education Scotland meetings and the Scottish Peer Education Network annual meeting. We intend to develop a public engagement activity as a spin-off from the intervention, and we will ensure that key results from papers are released to the media as appropriate. Peer supporters at participating schools will be supported to present on their experiences at practitioner dissemination meetings. Details of the study will be maintained on the University of Glasgow website. The full study protocol will be made publicly available through the NIHR website.

### Department of Health and Social Care disclaimer

The views expressed are those of the author(s) and not necessarily those of the NHS, the NIHR or the Department of Health and Social Care.

## Abbreviations

ASSIST: A Stop Smoking In School Trial
CHU9D: Child Health Utility 9 dimensions
CSO: Chief Scientist Office
DMEC: Data Monitoring and Ethics Committee
DMS: Designated Member of Staff
ICT: Information and communication technologies
IMD: Index of Multiple Deprivation
ISRCTN: International Standard Randomised Controlled Trial Number
IT: Information technology
MRC: Medical Research Council
NHS: National Health Service
NIHR-PHR: National Institute of Health Research Public Health Research
PI: Principal investigator
PPI: Patient and public involvement
QALY: Quality-adjusted life years
QoL: Quality of life
RCT: Randomised controlled trial
SCT: Social Cognitive Theory
SDT: Self-determination Theory
SHARE: Sexual Health and Relationships Education
SNT: Social Norms Theory
SPHSU: Social and Public Health Sciences Unit
SPIRIT: Standard Protocol Items: Recommendations for Interventional Trials
SRT: Self-regulation Theory
STAND: Students Together Against Negative Decisions
STASH: STis and Sexual Health
STI’s: Sexually transmitted infections
SWEMWBS: Short Warwick-Edinburgh Mental Well-being Scale
TSC: Trial Steering Committee
